# Systemic Immune-Inflammation Index Is a Prognostic Predictor in Patients with Intrahepatic Cholangiocarcinoma Undergoing Liver Transplantation

**DOI:** 10.1155/2021/6656996

**Published:** 2021-02-15

**Authors:** Ao Ren, Zhongqiu Li, Pengrui Cheng, Xuzhi Zhang, Ronghai Deng, Yi Ma

**Affiliations:** ^1^Organ Transplant Center, The First Affiliated Hospital, Sun Yat-sen University, Guangzhou, China; ^2^Guangdong Provincial Key Laboratory of Organ Donation and Transplant Immunology, The First Affiliated Hospital, Sun Yat-sen University, Guangzhou, China; ^3^Guangdong Provincial International Cooperation Base of Science and Technology (Organ Transplantation), The First Affiliated Hospital, Sun Yat-sen University, Guangzhou, China

## Abstract

**Background:**

It was reported that systemic immune inflammation index (SII) was related to poor prognosis in a variety of cancers. We aimed to investigate the ability of the prognostic predictors of SII in patients with intrahepatic cholangiocarcinoma (iCCA) undergoing liver transplantation (LT).

**Methods:**

The 28 iCCA patients who underwent LT at our hospital between 2013 and 2018 were reviewed. Kaplan–Meier survival curves and Cox regression analyses were used to evaluate the prognostic significance of SII. Patients were divided into the high and low SII groups according to the cut-off value.

**Results:**

The 1-, 3-, and 5-year OS rates were significantly lower in the high SII group (85.7%, 28.6%, and 21.4%, respectively) than in the low SII group (92.9%, 71.4%, and 57.2%, respectively; *P* = 0.009). The 1-, 3-, and 5-year RFS rates were, respectively, 57.1%, 32.7%, and 21.8% in the high SII group and 85.7%, 61.1%, and 61.1% in the low SII group (*P* = 0.021). SII ≥ 447.48 × 10^9^/L (HR 0.273, 95% CI 0.082–0.908; *P* = 0.034) was an independent prognostic factor for OS.

**Conclusions:**

Our results showed that SII can be used to predict the survival of patients with iCCA who undergo LT.

## 1. Introduction

Cholangiocarcinoma (CCA) is a rare malignant tumor with an incidence of less than 2/100,000 persons [1]. CCA is classified into several subtypes. iCCA accounts for 8–10% of biliary tract cancers [2]. The incidence of iCCA has been increasing worldwide over the last 3 decades, which may be related to primary sclerosing cholangitis, viral hepatitis, or chemical exposure [3]. Because of the poor long-term outcomes, iCCA is usually a contraindication for LT [4]. However, data from several studies have reported poor outcomes in patients with iCCA after transplantation [5]. Thus, many centers consider iCCA to be a contraindication to liver transplantation typically [6]. Despite the controversy, several studies have proposed that LT may provide acceptable long-term survival in selected patients with iCCA [7, 8]. Therefore, it is necessary to establish appropriate criteria to select the right patients for liver transplantation. The current criteria for evaluating liver transplantation, such as the Milan criteria and Hangzhou criteria, that are effective for hepatocellular carcinoma (HCC) patients are not useful for evaluating patients with iCCA.

There is sufficient evidence that inflammation is related to tumor progression [9, 10]. It was reported that inflammatory cells such as lymphocytes and platelets change the tumor microenvironment play an important role in promoting the proliferation, invasion, and migration of tumors. Inflammation-based scores, including PLR, PNI, and SII, have been reported to be useful prognostic biomarkers for various cancers [11–14]. SII has been proved to be a prognostic predictor for several cancers. However, it remains unclear whether there is a correlation between preoperative SII and prognosis in patients with iCCA undergoing LT. The purpose of this study was to explore the prognostic value of SII in patients with iCCA undergoing LT.

## 2. Methods

The 28 patients who received liver transplantation for iCCA at the First Affiliated Hospital, Sun Yat-Sen University (Guangzhou, China), from 2013 to 2018 were retrospectively reviewed. The diagnosis was confirmed by medical imaging and pathological examination of tissue specimens. Clinical characteristics extracted from the medical records. Patients were followed monthly for the first 6 months. This study only included patients with iCCA at the explant. Patients with mixed iCCA + HCC (in the same or different nodule) were excluded from the study.

All tumor patients including iCCA on the waiting list evaluated for extrahepatic metastasis were evaluated. Patients with an expected waiting list time of over 6 months could have been treated with transarterial chemoembolization (TACE), ablation as a bridge to LT. In addition to TACE and ablation, patients with iCCA diagnosed preoperatively received chemotherapy based on gemcitabine and cisplatin.

Independent *χ*^2^ tests were used to compare categorical variables. Continuous variables were compared using *t*-tests. Survival curves were analyzed using the Kaplan-Meier method. The Cox regression analysis was used for univariate and multivariate analyses. The area under the receiver operating characteristic (ROC) curve (AUC) was calculated to examine the predictive value of the proposed model. All statistical analyses were performed using SPSS version 19.0 statistical software (SPSS, Chicago, IL, USA). *P* values < 0.05 were considered statistically significant.

All organs came from voluntary donations from citizens; no organs from executed prisoners (even with his/her consent) were used involved. The study was approved by the Institutional Review Board of the First Affiliated Hospital of Sun Yat-sen University and was performed in accordance with the Declaration of Istanbul. All protocols conformed to the ethical guidelines of the 1975 Helsinki Declaration.

## 3. Results

A total of 28 consecutive adult liver transplant patients with iCCA were included in the analysis. Clinical characteristics are summarized in Table 1. Patients diagnosed with iCCA received adjuvant therapy, with gemcitabine and cisplatin, and only a subset of patients received TACE and ablation for pretransplant locoregional therapy.

The 28 patients in the study were 25 (%) male and 3 (%) females. The median age was 51.5 (interquartile range (IQR) 46.8–60.0) years. The median follow-up duration was 33.5 months. The 5-year OS rate was 39.3%, and the 5-year RFS rate was 43.0%, respectively.

The ROC curves of SII, NLR, and PLR indicated that 447.48, 2.92, and 106.62 were the optimal cut-off values. According to the cut-off values, patients were divided into the low (<447.48 × 10^9^/L, *n* = 14) and high (≥447.48 × 10^9^/L, *n* = 14) SII groups. The demographic and clinicopathological characteristics of the two groups were compared (Table 2).

**Figure 1:**
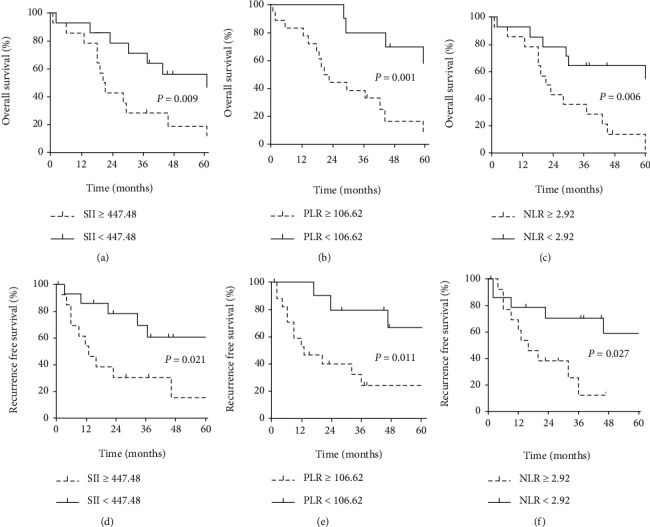
Overall survival curves after LT for iCCA patients classified by (a) SII, (b) PLR, and (c) NLR; recurrence-free survival curves after LT for iCCA patients classified by (d) SII, (e) PLR, and (f) NLR.

The 5-year OS rates were significantly lower in the high SII group than in the low SII group (21.4% vs. 57.2%, *P* = 0.009) (Figure 1(a)). The 5-year RFS rates were 21.8% in the high SII group and 61.1% in the low SII group (*P* = 0.021) (Figure 1(d)). High PLR and NLR scores were also associated with poor OS (*P* = 0.001 and *P* = 0.006; Figures 1(b) and 1(c)) and poor RFS (*P* = 0.011 and *P* = 0.027; Figures 1(e) and 1(f)).

Univariate analysis revealed CEA level, tumor recurrence, SII, PLR, and NLR to be significant prognostic factors for OS. Results of multivariate analysis showed that SII ≥ 447.48 × 10^9^/L was revealed to be an independent predictor of OS after LT in patients with iCCA (hazard ratio (HR) 0.273, 95% confidence interval (CI) 0.082–0.908; *P* = 0.034) (Table 3).

## 4. Discussion

Compared with HCC, iCCA has a higher recurrence rate and a worse prognosis. As such, liver transplantation for iCCA is highly controversial. Because of high recurrence rates and poor long-term survival, liver transplantation for iCCA has been abandoned in most transplantation centers. While the indications for liver transplantation for iCCA are controversial. In 2016, Sapisochin et al. [15] conducted a multicenter study that the advanced group had a higher 5-year recurrence rate than the very early iCCA group (61% vs. 15%, respectively) and lower 5-year OS (45% vs. 65%, respectively). Therefore, appropriate selection criteria are required to ensure a better prognosis of patients undergoing liver transplantation for iCCA. At present, there is no relevant study to explore the predictive value of SII in patients with iCCA for LT. In this study, patients who underwent LT for iCCA and demonstrated that a high SII (≥447.48 × 10^9^/L) significantly correlated with poorer prognosis.

As mentioned earlier, some studies have shown that inflammation factors are associated with prognosis in patients with cancer [16–18]. SII is widely accepted to be a new predictive marker to predict the prognosis of several types of cancer [19–21]. However, there are few studies on the prognosis of SII and iCCA. Although SII has been confirmed to be related to the prognosis of iCCA, the mechanism is not clear. SII is a systemic inflammatory marker, which can predict the prognosis of tumor from the level of inflammatory and immune. It has been reported that poor prognosis are concomitant with some inflammatory markers, such as NLR and PLR [22, 23]. Gomez et al. and Chen et al. reported that iCCA patients with a high preoperative NLR are related to poor prognosis [22, 24]. Chen et al. also confirmed that high PLR was related to poor prognosis [25].

The number of neutrophils in patients with malignant tumors increases plays an important role in the development of tumors [26–28]. It is reported that lymphocytes can mediate tumor regression effectively. The mechanism of which was realized by secreting cytokines and inducing cytotoxic cell death [29, 30]. In patients with intrahepatic cholangiocarcinoma, elevated NLR was independently associated with poor prognosis [31]. Platelets and neutrophils can secrete vascular endothelial growth factor (VEGF), which is important in tumor progression [32]. It has been shown that tumors are infiltrated by various lymphocytes, which is related to the progress of tumor [33, 34]. Immunooncology has become a promising approach in the field of new anticancer drug development [35, 36]. PD-L1 and HHLA2 are potential immunotherapeutic targets for iCCA patients [37, 38].

This study has several limitations. First, this was a retrospective, single-center analysis with a small number of patients. Second, SII was a dynamic index in the process of treatment and could be affected by unidentified infection and hepatitis B infection and so on.

In conclusion, our study suggests that preoperative SII is a simple and useful predictor of prognosis, which will help to select more suitable iCCA patients for liver transplantation and improve the prognosis of patients with cholangiocarcinoma after liver transplantation.

## Figures and Tables

**Table 1:** Baseline characteristics in iCCA patients.

Characteristic	Values
Gender	
Male	25 (89.3)
Female	3 (10.7)
Age (years)	51.5 (46.8-60.0)
Child-Pugh Class	
A	7 (25.0)
B	13 (46.4)
C	8 (28.6)
BMI	23.2 (20.4-23.9)
MELD score	11.0 (7.8-16.5)
CEA (*μ*g/L)	3.2 (2.5-8.6)
CA19-9 (U/L)	125.9 (28.1-1436.6)
AFP (ng/L)	4.2 (2.6-5.1)
Tumor number	
Single	19 (67.9)
Multiple	9 (32.1)
Largest tumor size (cm)	5.8 (2.4-7.5)
HBsAg	
Positive	17 (60.7)
Negative	11 (39.3)
Pretransplant locoregional therapy	21 (75.0)
Differentiation	
Well	2 (7.1)
Moderate	18 (64.3)
Poor	8 (28.6)
Vascular invasion	9 (32.1)
Tumor recurrence	15 (53.6)
SII	447.5 (289.9-930.7)
PLR	125.8 (98.6-184.1)
NLR	2.9 (1.8-4.1)
Follow-up (months)	33.5 (18.8-50.8)

Data are presented as *n* (%) or median (IQR).

**Table 2:** Comparison of characteristics between the high SII group and low SII group of patients with iCCA who underwent LT.

Variables	SII < 447.48 (*n* = 14)	SII ≥ 447.48 (*n* = 14)	*P* value
Gender			*P* = 0.541
Male	13 (92.9)	12 (85.7)	
Female	1 (7.1)	2 (14.3)	
Age (years)	50.0 (45.3-60.0)	54 (47.8-59.3)	*P* = 0.380
Child-Pugh Class			*P* = 0.242
A	4 (28.6)	3 (21.4)	
B	8 (57.1)	5 (35.7)	
C	2 (14.3)	6 (42.9)	
BMI	23.5 (22.4-25.0)	22.0 (19.9-23.5)	*P* = 0.423
MELD score	10 (7-14)	12 (8-18)	*P* = 0.592
CEA (*μ*g/L)	2.6 (1.4-4.2)	8.2 (3.1-19.3)	*P* = 0.321
CA19-9 (U/L)	59.6 (12.0-235.1)	576.5 (9.2-3874.4)	*P* = 0.264
AFP (ng/L)	3.2 (2.6-5.6)	4.3 (2.8-5.5)	*P* = 0.624
Tumor number			*P* = 0.686
Single	10 (71.4)	9 (64.3)	
Multiple	4 (28.6)	5 (35.7)	
Largest tumor size (cm)	2.9 (2.0-7.6)	6.1 (4.3-7.0)	*P* = 0.428
HBsAg			*P* = 0.699
Positive	8 (57.1)	9 (64.3)	
Negative	6 (42.9)	5 (35.7)	
Pretransplant locoregional therapy	11 (78.6)	10 (71.4)	*P* = 0.663
Differentiation			*P* = 0.329
Well	2 (14.3)	0 (0.0)	
Moderate	8 (57.1)	10 (71.4)	
Poor	4 (28.6)	4 (28.6)	
Vascular invasion	3 (21.4)	5 (35.7)	*P* = 0.403
Tumor recurrence	6 (42.9)	9 (64.3)	*P* = 0.256

Data are presented as *n* (%) or median (IQR).

**Table 3:** Univariate and multivariate analyses of factors related to overall survival in patients with iCCA who underwent LT.

Variables	Univariate analysis			Multivariate analysis		
	HR	95% CI	*P* value	HR	95% CI	*P* value
Gender (male vs. female)	1.452	0.422-4.997	0.554			
Age (years)	1.035	0.989-1.082	0.142			
Child-Pugh Class (A or B vs. C)	1.579	0.629-3.964	0.331			
BMI	0.960	0.823-1.120	0.605			
MELD score	1.014	0.974-1.056	0.508			
HBsAg (positive vs. negative)	1.336	0.531-3.359	0.538			
Differentiation (well or moderate vs. poor)	1.724	0.711-4.180	0.228			
AFP (ng/mL) (>20 vs. ≤20)	1.656	0.217-12.66	0.627			
CEA (*μ*g/L) (>10 vs. ≤10)	0.292	0.091-0.937	0.038	0.713	0.170-2.997	0.645
CA19-9 (U/L) (>37 vs. ≤37)	0.742	0.295-1.868	0.526	1.202	0.406-3.564	0.740
Largest tumor size (cm) (>5 vs. ≤5)	0.399	0.157-1.011	0.053	0.689	0.192-2.470	0.567
Tumor number (multiple vs. single)	1.309	0.519-3.299	0.569	1.653	0.493-5.550	0.416
Pretransplant locoregional therapy (yes vs. no)	1.218	0.467-3.177	0.687	0.760	0.214-2.698	0.671
Vascular invasion (yes vs. no)	0.543	0.197-1.496	0.237	0.547	0.164-1.823	0.326
Tumor recurrence (yes vs. no)	5.101	1.672-15.569	0.004	3.106	0.723-13.338	0.127
SII (≥447.48)	0.311	0.122-0.792	0.014	0.273	0.082-0.908	0.034
PLR (≥106.62)	0.188	0.061-0.582	0.004	0.313	0.075-1.314	0.113
NLR (≥2.92)	0.269	0.099-0.729	0.01	0.496	0.146-1.689	0.262

## Data Availability

The data used to support the findings of this study have not been made available because of local ethical guidelines.

## References

[1] Bergquist A., von Seth E. (2015). Epidemiology of cholangiocarcinoma. *Best Practice & Research. Clinical Gastroenterology*.

[2] Gupta A., Dixon E. (2017). Epidemiology and risk factors: intrahepatic cholangiocarcinoma. *Hepatobiliary Surgery and Nutrition*.

[3] Vogel A., Saborowski A. (2017). Cholangiocellular carcinoma. *Digestion*.

[4] Mazzaferro V., Gorgen A., Roayaie S., Droz dit Busset M., Sapisochin G. (2020). Liver resection and transplantation for intrahepatic cholangiocarcinoma. *Journal of Hepatology*.

[5] Laurent S., Verhelst X., Geerts A. (2019). Update on liver transplantation for cholangiocarcinoma : a review of the recent literature. *Acta Gastroenterologica Belgica*.

[6] Bridgewater J., Galle P. R., Khan S. A. (2014). Guidelines for the diagnosis and management of intrahepatic cholangiocarcinoma. *Journal of Hepatology*.

[7] Goldaracena N., Gorgen A., Sapisochin G. (2018). Current status of liver transplantation for cholangiocarcinoma. *Liver Transplantation*.

[8] Takahashi K., Obeid J., Burmeister C. S. (2016). Intrahepatic cholangiocarcinoma in the liver explant after liver transplantation: histological differentiation and prognosis. *Annals of Transplantation*.

[9] Grivennikov S. I., Greten F. R., Karin M. (2010). Immunity, inflammation, and cancer. *Cell*.

[10] Diakos C. I., Charles K. A., McMillan D. C., Clarke S. J. (2014). Cancer-related inflammation and treatment effectiveness. *The Lancet Oncology*.

[11] Mowbray N. G., Griffith D., Hammoda M., Shingler G., Kambal A., al-Sarireh B. (2018). A meta-analysis of the utility of the neutrophil-to-lymphocyte ratio in predicting survival after pancreatic cancer resection. *HPB: The Official Journal of the International Hepato Pancreato Biliary Association*.

[12] Pang Q., Zhang L. Q., Wang R. T. (2015). Platelet to lymphocyte ratio as a novel prognostic tool for gallbladder carcinoma. *World Journal of Gastroenterology*.

[13] Qi X., Li J., Deng H., Li H., Su C., Guo X. (2016). Neutrophil-to-lymphocyte ratio for the prognostic assessment of hepatocellular carcinoma: a systematic review and meta-analysis of observational studies. *Oncotarget*.

[14] Sellers C. M., Uhlig J., Ludwig J. M., Stein S. M., Kim H. S. (2019). Inflammatory markers in intrahepatic cholangiocarcinoma: effects of advanced liver disease. *Cancer Medicine*.

[15] Sapisochin G., Facciuto M., Rubbia-Brandt L. (2016). Liver transplantation for “very early” intrahepatic cholangiocarcinoma: international retrospective study supporting a prospective assessment. *Hepatology*.

[16] Suzuki Y., Okabayashi K., Hasegawa H. (2018). Comparison of preoperative inflammation-based prognostic scores in patients with colorectal cancer. *Annals of Surgery*.

[17] Nakagawa K., Sho M., Akahori T. (2019). Significance of the inflammation-based prognostic score in recurrent pancreatic cancer. *Pancreatology*.

[18] Yamamoto M., Kobayashi T., Kuroda S. (2019). Verification of inflammation-based prognostic marker as a prognostic indicator in hepatocellular carcinoma. *Annals of Gastroenterological Surgery*.

[19] Holub K., Biete A. (2019). Impact of systemic inflammation biomarkers on the survival outcomes of cervical cancer patients. *Clinical & Translational Oncology*.

[20] Murthy P., Zenati M. S., al Abbas A. I. (2020). Prognostic value of the systemic immune-inflammation index (SII) after neoadjuvant therapy for patients with resected pancreatic cancer. *Annals of Surgical Oncology*.

[21] Berardi R., Santoni M., Rinaldi S. (2019). Pre-treatment systemic immune-inflammation represents a prognostic factor in patients with advanced non-small cell lung cancer. *Annals of Translational Medicine*.

[22] Chen Q., Yang L. X., Li X. D. (2015). The elevated preoperative neutrophil-to-lymphocyte ratio predicts poor prognosis in intrahepatic cholangiocarcinoma patients undergoing hepatectomy. *Tumour Biology*.

[23] Lin J., Fang T., Zhu M. (2019). Comparative performance of inflammation-based prognostic scores in patients operated for intrahepatic cholangiocarcinoma. *Cancer Management and Research*.

[24] Gomez D., Morris-Stiff G., Toogood G. J., Lodge J. P. A., Prasad K. R. (2008). Impact of systemic inflammation on outcome following resection for intrahepatic cholangiocarcinoma. *Journal of Surgical Oncology*.

[25] Chen Q., Dai Z., Yin D. (2015). Negative impact of preoperative platelet-lymphocyte ratio on outcome after hepatic resection for intrahepatic cholangiocarcinoma. *Medicine (Baltimore)*.

[26] Elinav E., Nowarski R., Thaiss C. A., Hu B., Jin C., Flavell R. A. (2013). Inflammation-induced cancer: crosstalk between tumours, immune cells and microorganisms. *Nature Reviews. Cancer*.

[27] Coffelt S. B., Wellenstein M. D., de Visser K. E. (2016). Neutrophils in cancer: neutral no more. *Nature Reviews. Cancer*.

[28] Petrie H. T., Klassen L. W., Kay H. D. (1985). Inhibition of human cytotoxic T lymphocyte activity in vitro by autologous peripheral blood granulocytes. *Journal of Immunology*.

[29] Robbins P. F. (2017). Tumor-infiltrating lymphocyte therapy and neoantigens. *Cancer Journal*.

[30] Ferrone C., Dranoff G. (2010). Dual roles for immunity in gastrointestinal cancers. *Journal of Clinical Oncology*.

[31] Buettner S., Spolverato G., Kimbrough C. W. (2018). The impact of neutrophil-to-lymphocyte ratio and platelet-to-lymphocyte ratio among patients with intrahepatic cholangiocarcinoma. *Surgery*.

[32] Bambace N. M., Holmes C. E. (2011). The platelet contribution to cancer progression. *Journal of Thrombosis and Haemostasis*.

[33] Chew V., Chen J., Lee D. (2012). Chemokine-driven lymphocyte infiltration: an early intratumoural event determining long-term survival in resectable hepatocellular carcinoma. *Gut*.

[34] West N. R., Kost S. E., Martin S. D. (2013). Tumour-infiltrating FOXP3(+) lymphocytes are associated with cytotoxic immune responses and good clinical outcome in oestrogen receptor-negative breast cancer. *British Journal of Cancer*.

[35] Robert C., Schachter J., Long G. V. (2015). Pembrolizumab versus ipilimumab in advanced melanoma. *The New England Journal of Medicine*.

[36] Garon E. B., Rizvi N. A., Hui R. (2015). Pembrolizumab for the treatment of non-small-cell lung cancer. *The New England Journal of Medicine*.

[37] Jing C. Y., Fu Y. P., Yi Y. (2019). HHLA2 in intrahepatic cholangiocarcinoma: an immune checkpoint with prognostic significance and wider expression compared with PD-L1. *Journal for Immunotherapy of Cancer*.

[38] Mody K., Starr J., Saul M. (2019). Patterns and genomic correlates of PD-L1 expression in patients with biliary tract cancers. *Journal of Gastrointestinal Oncology*.

